# Synthesis and Characterization of Linear Polyisoprene Supramolecular Elastomers Based on Quadruple Hydrogen Bonding

**DOI:** 10.3390/polym12010110

**Published:** 2020-01-05

**Authors:** Yaoke Ding, Jincheng Wang, Shiqiang Song

**Affiliations:** College of Chemistry and Chemical Engineering, Shanghai University of Engineering Science, Shanghai 201620, China; dingyaoke0604@163.com (Y.D.); songchem@126.com (S.S.)

**Keywords:** supramolecular elastomer, synthesis, UPy group, properties

## Abstract

Supramolecular elastomers based on quaternary hydrogen bonding of ureido-pyrimidinone (UPy) groups own special properties such as reversibility, self-healing, and good processability, which can be used in many special fields. In this paper, a novel type of linear polyisoprene supramolecular elastomer (LPSE) was prepared via anionic polymerization by deliberately introducing hydroxyl, isocyanate, and UPy groups into the ends. The formation of supramolecular structure showed significant effects on the microphase structures of LPSE, which was characterized by Fourier-transform infrared spectroscopy (FTIR), gel permeation chromatography (GPC), hydrogen nuclear magnetic resonance (^1^H-NMR), and dynamic mechanical analysis (DMA). Results showed that the introduction of UPy groups played a certain role in the improvement of the thermal stability, toughness, and tensile strength of the elastomer. Moreover, from self-healing tests, the hydrogen bonds of UPy showed dynamic characteristics which were different from covalent sacrificial bonds and exhibited the reassociation phenomenon. This study can not only extend our understanding of the toughening effect of strong hydrogen bonds, but also help us to rationally design new and tough elastomers.

## 1. Introduction

In recent years, with the rapid development of the rubber industry, the supply of elastomers became tight and the price became high. The market demand for isoprene rubber also increased [1]. However, for rubber polymers such as polyisoprene, it is difficult to recycle and reuse them in the industry. Hence, the materials used there must be sustainable during the life cycle and must be self-healing in the event of minor damage during this service. Therefore, research on the development of new polyisoprene polymers, especially those with thermoreversibility and self-healing properties, is of great significance to meet the needs of materials processing technology.

Supramolecular polymers, as a type of novel materials, were widely researched recently [2]. These polymers combine supramolecular chemistry and polymer science, and they possess many unique characteristics. They have potential applications in many fields, such as self-healing materials [3,4], degradable drug nanocarriers [5,6], heterogeneous catalysis [7,8], molecular muscles [9,10], and stimuli-responsive supramolecular gels [11,12]. Among them, supramolecular elastomers, which require good processing properties at elevated temperatures, attracted great attention worldwide [13,14]. 

In the past few decades, research on supramolecular polymerization progressed greatly from spontaneous to controlled polymerization and living supramolecular polymerization [15,16]. The interactions among supramolecular polymers feature many types, such as hydrogen bonds, π–π interactions, hydrophobic interactions, and so on [17,18]. As one of them, the hydrogen bonding network system features medium strength, high directivity, and selectivity, and it makes up the most important and popular secondary interactions in constructing supramolecular structures.

However, the hydrogen bonds are weaker compared to covalent and ionic bonds. Therefore, if a polymer is to enhance its performance by hydrogen bonding, a plurality of hydrogen bonding interactions may be introduced to form hydrogen bonding networks. In this way, the 2-ureido-4 [1*H*]-pyrimidinone (UPy) unit exhibits a very broad application prospect. It can increase the strength of hydrogen bonds by forming dimers and quadruple hydrogen bonds which require higher energy to be destroyed. Therefore, the energy dissipation and toughness of the elastomer can be increased by introducing multiple hydrogen bonds [19]. Hydrogen bonding systems based on UPy units were used to prepare supramolecular polymers with a variety of polymer monomers, including polypropylenes [20], poly(methyl methacrylate)s, poly(ethlene glycol)s [21], tetraphenylethenes [22], poly(dimethyl siloxane)s [23], poly(butadiene)s [24], and so on. 

**Figure 1:**
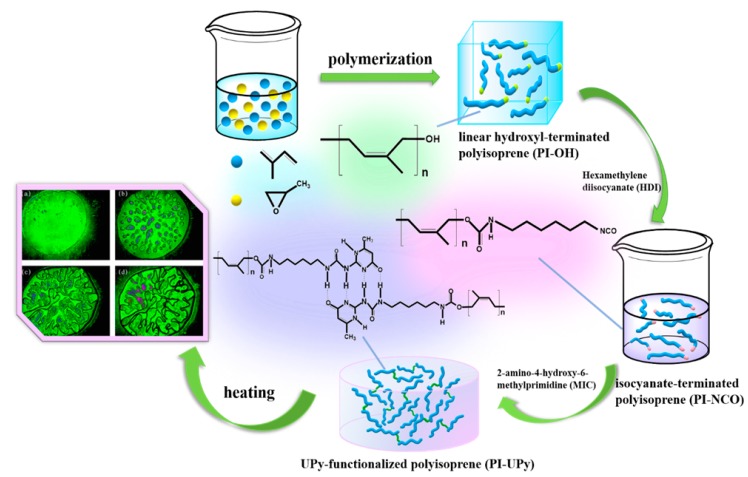
Synthetic process of 2-ureido-4 [1*H*]-pyrimidinone (UPy)-functionalized linear polyisoprene supramolecular elastomer (LSPE) and its heating photos by polarizing microscope (POM).

Inspired by the above work, we fabricated a self-healing linear supramolecular elastomer (LSPE) by virtue of multiple hydrogen bonds and bulk anionic polymerization (Figure 1). Here, the synthetic route containing the interaction of hydrogen bonds was provided. Bulk polymerization was used to get high-molecular-weight polymers, and polyisoprene with a termination –OH(PI-OH) was obtained via this method [25]. UPy was used to supply quadruple hydrogen bonding to improve the material performance for the modification of polyisoprene. Here, PI-UPy was prepared through the reaction between –NCO and 2-amino-4-hydroxy-6-methylprimidine (MIC). The polymer with UPy features high thermal stability and good mechanical properties. Meanwhile, the presence of hydrogen bonds can make polymers exhibit better self-healing performance. This property gives the UPy system-based supramolecular polymers application potential in some fields.

## 2. Experimental Methods

### 2.1. Materials

Isoprene (TCI, Tokyo, Japan, 99%) was freshly distilled and stored over a molecular sieve of 4Å prior to polymerization. Propylene oxide (Adamas, Shanghai, China, AR), *sec*-butyllithium (Energy, Shanghai, China, 1.3M in Hexane), 2-amino-4-hydroxy-6-methylprimidine (MIC) (Adamas, Shanghai, China, 98%+), and isopropyl alcohol (Energy, Shanghai, China, AR) were used as received. Methanol (Adamas, Shanghai, China, AR) was used after degassing. Hexamethylene diisocyanate (HDI, 99%) and dibutyl tin dilaurate (DBDTL, 95%+) were purchased from Adamas (Shanghai, China). Chloroform (CHCl_3_, ≥99%) was purchased from Greagent (Shanghai, China).

### 2.2. Synthesis of Linear Hydroxyl-Terminated Polyisoprene (PI-OH)

Isoprene was immersed in a molecular sieve for more than 24 h to remove water before use. Then, 50 mL of isoprene was added into a three-necked flask equipped with a thermometer through a syringe (20 mL) under nitrogen atmosphere, and it was maintained below 10 °C by an ice-water bath. Next, 0.5 mL of *N*-butyllithium (n-BuLi) was added to the above solution using a syringe (1 mL) with a 0.9-mm stainless-steel needle to initiate polymerization, and a polymer was obtained with high molecular weight. The reaction was ensured to proceed for 2 h and quantitative conversion was possible. The liquid in the reaction vessel was observed to have a certain viscosity, and then 20 mL of propylene oxide was injected to graft the produced polymer, which was maintained at 40 °C for 4 h. Finally, 5 mL of methanol was ultrasonically degassed in an ultrasonic cleaner and was added to the flask to terminate the reaction. The product was poured into a beaker, and an excess of isopropanol was added to the product for precipitation three times to purify the polymer, which was then placed under high vacuum for further use. 

### 2.3. Synthesis of Isocyanate-Terminated Polyisoprene (PI-NCO)

Firstly, 12 g (0.0002 mol) of PI-OH was dissolved in 100 mL of chloroform. Then, the mixture was added to a nitrogen-filled three-necked flask, and three drops of DBDTL catalyst were injected. Next, 25 mL of HDI was poured into the solution, and it was magnetically stirred at room temperature (r.t.) under a nitrogen atmosphere for 1 h. The system was then heated to 50 °C and stirred for 5 h. Finally, the resulting white product (i.e., PI-NCO) was dried under high vacuum and was stored under a nitrogen atmosphere for further use.

### 2.4. Synthesis of UPy-Functionalized Polyisoprene (PI-UPy)

PI-UPy was prepared by a reaction between PI-NCO and MIC. Firstly, 12 g (0.0001 mol) of PI-NCO was dissolved in chloroform and added to a three-necked flask together with 0.0248 g (0.0002 mol) of MIC powder. The flask was then evacuated and filled with nitrogen, and the reaction was magnetically stirred at 40 °C for 4 h. Then, it was precipitated in a beaker to which excess methanol was added. Finally, the mixture was stirred and repeatedly precipitated three times, and the product was obtained after vacuum-drying at 50 °C. 

### 2.5. Characterization

Proton nuclear magnetic resonance (^1^H-NMR) spectra were tested on a Bruker Advance (Karlsruhe, Germany) in CDCl_3_ as deuterated solvent at r.t. The chemical shift at 7.26 ppm referred to the signal of residual CHCl_3_. The crosslinked polymer was measured by solid-state ^1^H-NMR(600 MHz) on a Bruker spectrometer. Fourier-transform infrared spectroscopy (FTIR) was used to analyze the structure of the polymer at r.t. using a conversion spectrometer model Thermo Fisher Nicolet AVATAR 370 (Waltham, MA, USA) with a resolution of 4 cm^−1^. All of the polymers were tested in attenuated total reflectance (ATR) mode. The measured wavenumbers ranged from 4000 to 400 cm^−1^. The molecular weight was determined by gel permeation chromatography BI-MwA (GPC, Milford, CT, USA) at a temperature of 35 °C. Tetrahydrofuran was selected as the mobile phase at a flow rate of 1.0 mL/min. Polystyrene was used as a reference material for calibration. X-ray diffraction (XRD) was conducted using a Germany Bruker D2 PHASERA (Karlsruhe, Germany). The X-ray beam was nickel-filtered Cu-Kα (λ = 0.154 nm) radiation and was operated at 50 kV. The scanning range was from 5° to 80° with a scanning rate of 2°/min. Thermogravimetric analysis (TGA) and TGA-IR were conducted on a TA Q600 SDT instrument (TA, New Castle, DE, USA) under N_2_ atmosphere in a range of 25 to 800 °C with a heating rate of 20 °C/min. The relaxation behavior was analyzed by dynamic mechanical analysis (DMA) on a Q800 (TA, New Castle, DE, USA) instrument, operating in a single cantilever mode at a frequency of 1 Hz. The heating rate was 3 °C/min in a range of −60 to 60 °C. Scanning electron microscopy (SEM) measurement was performed on an SU8010 200 kV equipment (Hitachi, Tokyo, Japan) with a potential of 5 kV. The samples were sprayed with a conductive gold layer before photographing. Photomicrographs were taken with a Tianxing XPF-300 polarizing microscope (POM, Shanghai, China), and the magnification was 400–1000×. Stress–strain measurements were performed on a TCS-2000 universal tensile testing machine (Gotech easting machines company, Taichung, Taiwan) at r.t. with a rate of 500 mm/min on samples that were cut into dumbbell shapes with a width of 4 mm and a measurement length of 2.5 cm. 

## 3. Results and Discussion

### 3.1. Synthetic Process Analysis of LPSE

**Figure 2:**
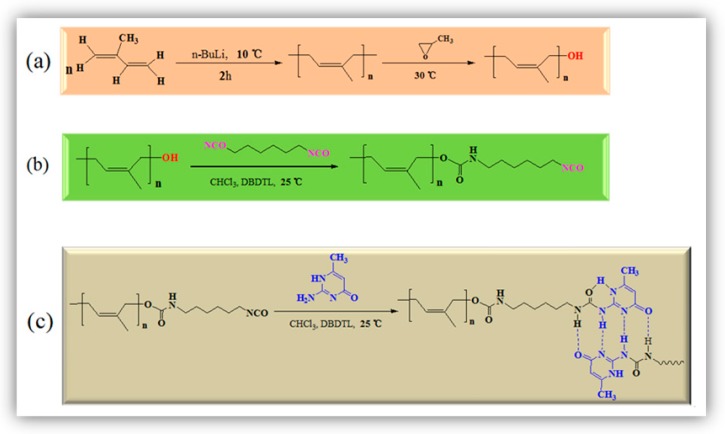
Synthesis of (**a**) linear hydroxyl-terminated polyisoprene (PI-OH), (**b**) isocyanate-terminated polyisoprene (PI-NCO), and (**c**) UPy-functionalized polyisoprene (PI-UPy).

Bulk anionic polymerization is a simple and direct method to synthesize polymeric materials with various structures. Figure 2a presents the preparation process of PI and PI-OH using the anionic polymerization method. In this experiment, in order to avoid the unsuccessful coupling reaction, some reaction conditions were optimized. Firstly, the inhibitors were removed by atmospheric distillation before the use of isoprene. Then, a 4-Å molecular sieve was used for removing the water in isoprene and propylene oxide. Thirdly, argon was used to purge the dissolved oxygen in the above materials. It was found that the coupling reaction can be successfully inhibited under such conditions. The formation of PI-NCO is given in Figure 2b. The aminomethyl ester was formed due to the reaction between –NCO in HDI and the terminal –OH in PI-OH. In addition, a substituted urea was formed in PI-UPy via a reaction between the terminal isocyanate groups in PI-NCO and amino groups in the structure of MIC (Figure 2c). 

### 3.2. Structure Analysis of LPSE

**Figure 3:**
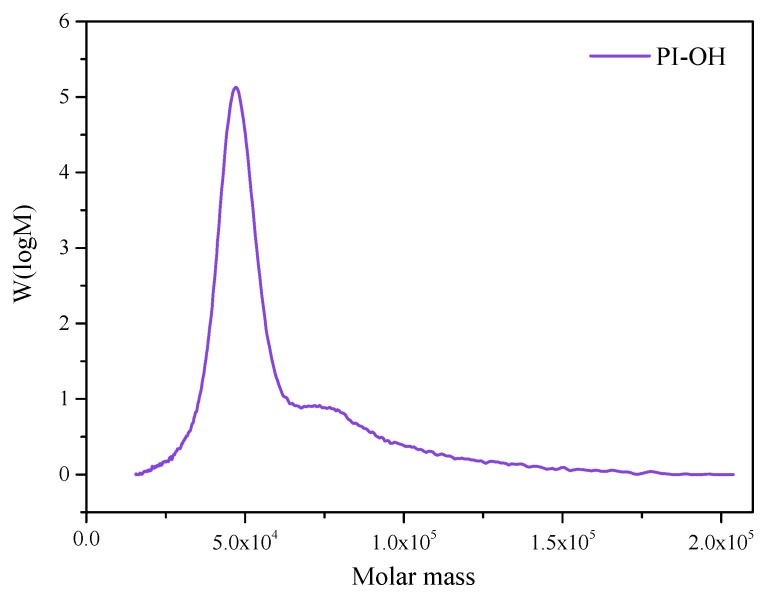
Molecular weight and its distribution of PI-OH.

The relative molecular mass of hydroxyl-terminated polyisoprene is an important indicator of its performance. GPC was used to analyze the molecular weight together with the distribution (*M*_w_/*M*_n_) of PI-OH. A functionalized polymer with diverse architecture may be obtained from anionic polymerization, which is a facile method to synthesize this type of macromolecule [14]. It can be seen from Figure 3 that PI-OH displayed a sharp peak, good symmetry, and a narrow molecular weight distribution, which helped to improve some properties of the polymer [26]. The symmetric and monomodal GPC curves with narrow *M*_w_*/M*_n_ confirmed the success of living anionic polymerization (Table 1) [27]. 

**Figure 4:**
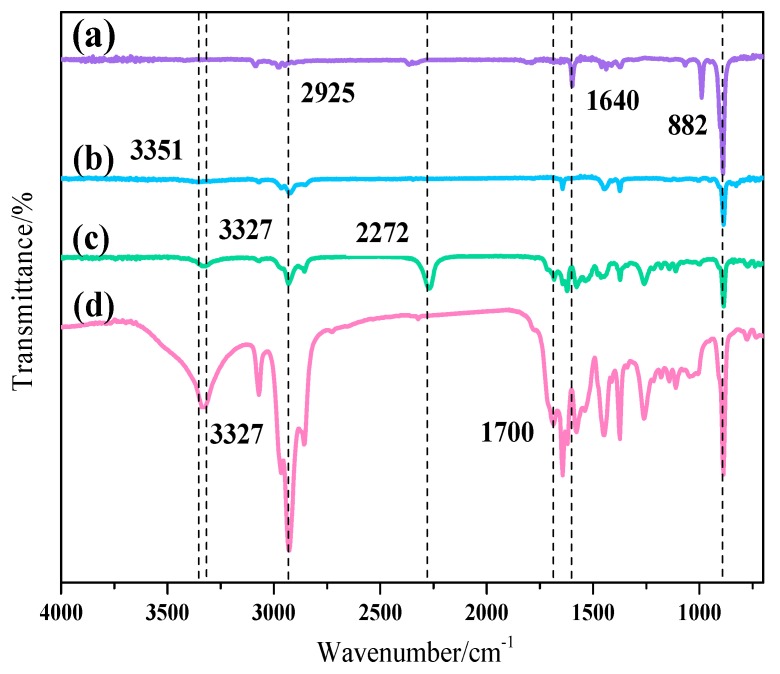
Fourier-transform infrared (FTIR) spectra of (**a**) isoprene, (**b**) PI-OH, (**c**) PI-NCO, and (**d**) PI-UPy.

The FTIR spectra of isoprene, PI-OH, PI-NCO, and PI-UPy are presented in Figure 4. In the FTIR spectrum of isoprene (Figure 4a), the characteristic peak at 2925 cm^−1^ was attributed to –CH_3_, and the absorption peak at 1640 cm^−1^ was derived from the stretching vibration of the double bonds. The stretching vibration at 882 cm^−1^ was related to the *cis*-3,4 chain structure in isoprene [28]. Compared with isoprene, the broad characteristic peak at 3351 cm^−1^ was related to the existence of hydroxyl groups in PI-OH (Figure 4b). For PI-NCO (Figure 4c), a strong absorption peak appeared at 2272 cm^−1^**,** which was attributed to the stretching vibration of –NCO in HDI. This illustrated that the active isocyanate groups existed in the product. The hydroxyl groups around at 3351 cm^−1^, together with –NH bonds at about 3327 cm^−1^, were observed [19]. In the spectrum of PI-UPy (Figure 4d), due to the successful reaction between MIC and PI-NCO, the characteristic peak at 2272 cm^−1^,disappeared and the absorption peak of –NH bonds appeared at 3327 cm^−1^. Moreover, the characteristic peaks at 1500–1700 cm^−1^ were ascribed to the bending vibrations of –NH bonds in UPy [29]. 

**Figure 5:**
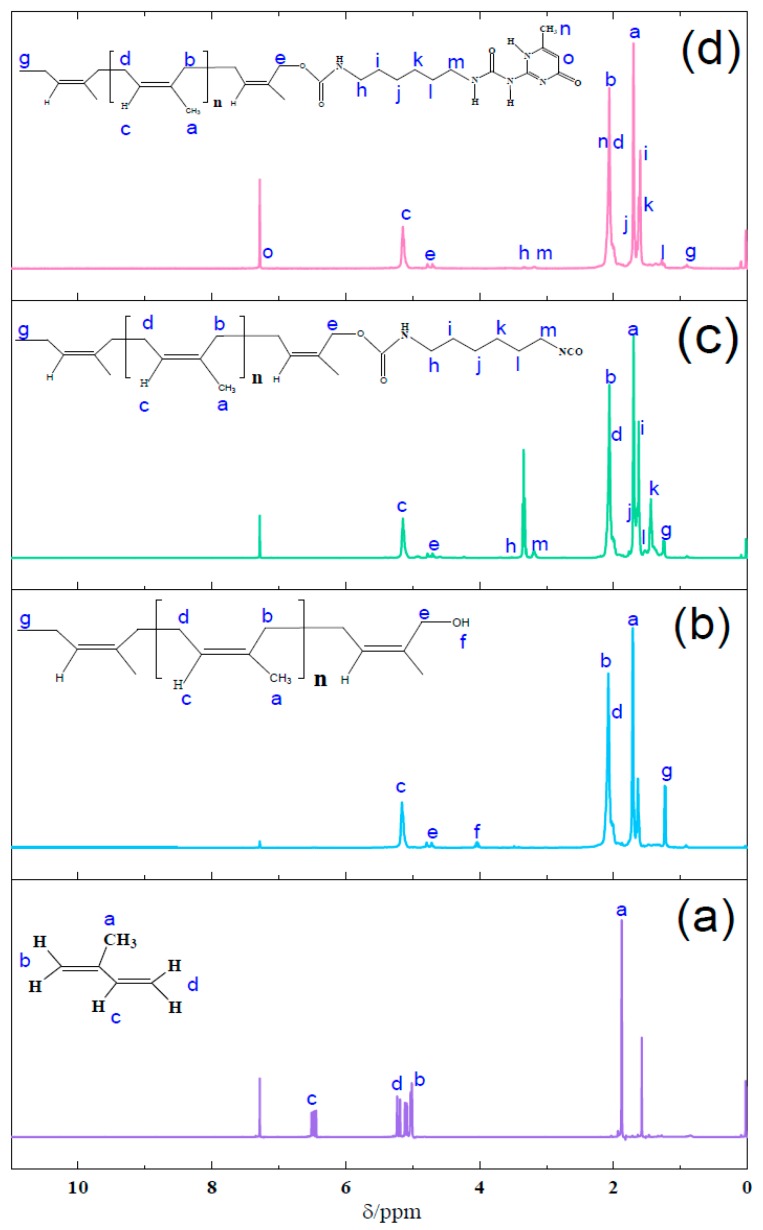
^1^H-NMR spectra of (**a**) isoprene, (**b**) PI-OH, (**c**) PI-NCO, and (**d**) PI-UPy.

The ^1^H-NMR spectra of isoprene, PI-OH, PI-NCO, and PI-UPy are given in Figure 5. In Figure 5a, the proton peak with a chemical shift at 1.88 ppm (peak a) was ascribed to the methyl group, and the peaks at 5.01–5.23 ppm (peaks b,d) referred to the methylene groups in isoprene. In Figure 5b, for PI-OH, the characteristic resonance peaks were exhibited at 1.71 (peak a), 2.07 and 4.75 (peak b and e), and 5.16 ppm (peak c), which were assigned to methyl, methylene, and C–H bonds, respectively [30]. Moreover, the characteristic resonance peak at 4.04 ppm (peak f) confirmed the formation of hydroxyl end groups. Figure 5c presents the ^1^H-NMR spectrum of PI-NCO. Compared with the spectrum of PI-OH, the chemical shift at 3.3–3.4 ppm (peak e) and other proton peaks at 1.24–1.69 and 1.92–3.41 ppm represented –CH_2_ in PI-NCO [31]. For PI-UPy (Figure 5d), peak n at 2.31 ppm belonged to the methyl groups, and 7.32 ppm (peak o) was the characteristic peak of –CH bonds in UPy. Proton signals of NH sometimes disappeared and showed chemical shifts depending on the solvent conditions.

### 3.3. Thermal Stability of LPSE

**Figure 6:**
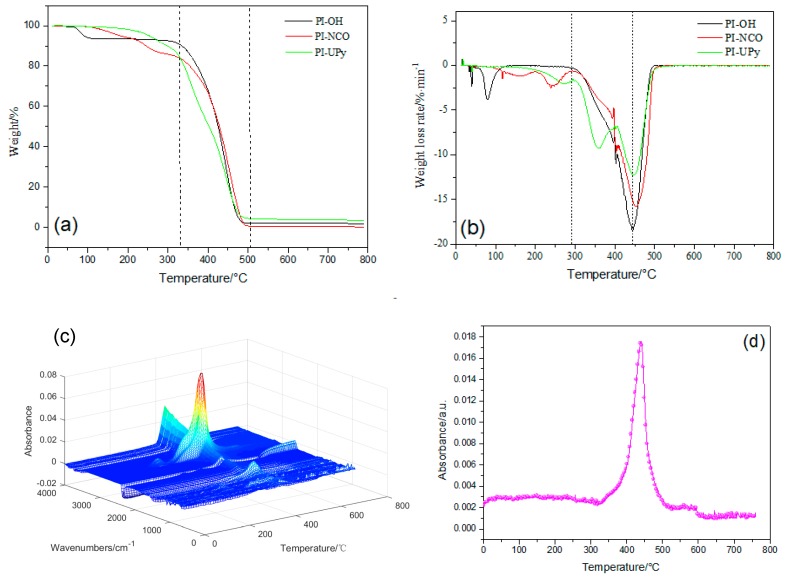
Thermogravimetric analysis (TGA) (**a**) and DTG (**b**) of PI-OH, PI-NCO, and PI-UPy; (**c**,**d**) TGA infrared (TGA-IR) data for PI-UPy.

PI-OH presented two stages of weight loss as revealed by TGA (Figure 6a). The first stage started at ~70 °C and was attributed to the degradation of excessive isoprene and non-volatile isopropanol. The second stage started at ~330 °C and resulted from the decomposition of polyisoprene segments [32]. For PI-NCO, it firstly degraded at ~100 °C, which was equivalent to a weight loss of 6%, and then the urea bonds cleaved at ~225 °C [33]. For PI-UPy, the first decomposition step appeared at 200 °C, and the second step was ascribed to the decomposition of polyisoprene. In addition, from the DTG curves (Figure 6b), the mass loss rate of decomposition between 300 and 400 °C was different, and PI-UPy showed the slowest decomposition behavior [26]. In summary, the addition of UPy facilitated the formation of intermolecular hydrogen bonds and, thus, the thermal stability of the polymer was improved.

Figure 6c presents the TGA-IR curves of PI-UPy. When the temperature was higher than 200 °C, some absorption peaks began to appear at 3500 cm^−1^ in the gas phase. This illustrated that H_2_O may appear in this stage. As considered, isocyanate and hydroxyl groups may be contained in this polymer, and this may be related to the decomposition of urethane and urea groups. With an increase in temperature, absorption peaks of different intensities appeared between 300 and 500 °C at various wavenumbers in the gas phase. In addition, the absorption peaks around 3300–3700 cm^−1^ were strong and reached the maximum level at 500 °C. This indicated that CH_4_, NH_3_, and H_2_O were generated from the decomposition of PI-UPy segments. Moreover, the absorption peaks around 3300 cm^−1^ always existed until the composite was completely degraded. In addition, a weak CO_2_ absorption peak around 2300 cm^−1^ appeared, and this resulted from the decomposition products, olefins, and alkanes.

### 3.4. Mechanical Properties of LPSE

**Figure 7:**
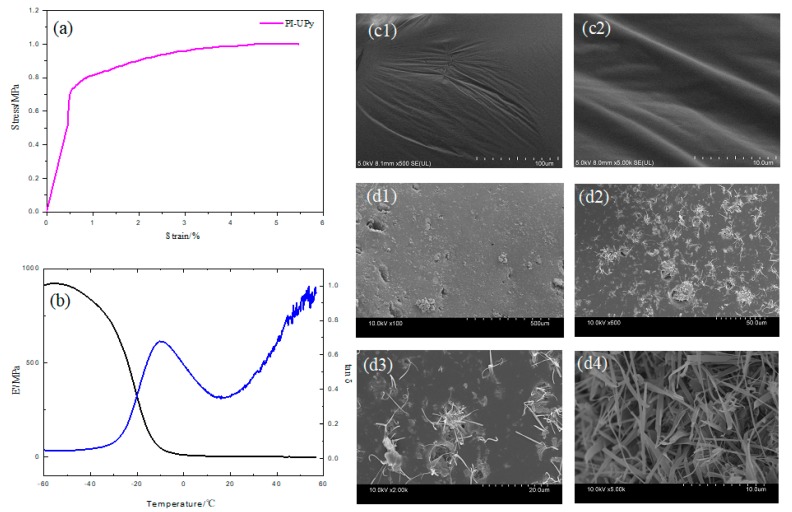
Mechanical behavior of PI-UPy: (**a**) stress–strain curves for PI-UPy; (**b**) E’ and tan δ; SEM of (**c**1–2) PI-OH and (**d**1–4) PI-UPy.

In the stress–strain curve of PI-UPy (Figure 7a), with the application of stress, a significant stress hardening phenomenon occurred after a certain deformation. The initial strain of this stress hardening was reduced due to the addition of the UPy groups. Typically, this phenomenon was caused by strain-induced crystallization (SIC) [26,34]. The length of the macromolecular chains of the elastomer may be increased by the presence of UPy groups and, thus, its elongation may be improved. The hydrogen bonds of the UPy groups in the elastomer did not permanently interact, and they dissociated and dissipated energy when the deformation energy was higher than that of the hydrogen bonds itself. Thus, during this process, the elastomer redistributed stress and caused further dissociation of hydrogen bonds throughout the sample. Therefore, the elastomer can withstand higher stresses after stress hardening and undergo greater deformation [35]. The trend of storage modulus E’ and loss factor tan δ with temperature is shown in Figure 7b. The elasticity and stiffness of the elastomer are characterized by the magnitude of E’ [29]. It can be seen from the black line that E’ began to decrease slightly when the temperature of the elastomer reached about −50 °C. As the temperature rose further, E’ dropped sharply to a constant value at a temperature of −10 °C. The possibility for movement of the molecular chains in all directions was limited due to the lack of free volume. Therefore, under load or pressure, the molecule cannot respond accordingly. As a result, the elastomer exhibits a high modulus value [32]. However, as the temperature gradually rose to near the glass transition temperature, the free volume of the segment also gradually increased, and this resulted in an increase in the mobility of the molecular segment. Under the application of load and stress, the sample produced greater strain due to the movement of the segment. The modulus of the sample was lowered within the temperature range between −50 and −10 °C, which was the glass transition temperature of PI-UPy [36]. The blue line shows the value of tan δ for PI-UPy. The curve of tan δ versus temperature showed a clear and symmetrical peak, which illustrated that its relaxation corresponded to the transition from the glassy state to the elastic state of the rubber. The peak appearing at −10 °C was related to the glass transition temperature of PI-UPy, that is, the characteristic temperature corresponding to the peak. When the temperature was higher than 20 °C, the tan δ value also obviously increased, and this indicated that its damping properties also increased. The surface morphology of PI-OH and PI-UPy was further revealed by SEM. The surface status of PI-OH was smooth and flat (Figure 7c1,2). After modification by UPy, filaments were formed on the material’s surface within a few days of incubation (Figure 7d1–4). They were homogeneously dispersed in the matrix, which was ascribed to the introduction of UPy into the system, and this can improve the tensile performance of the material [35]. 

### 3.5. Self-Healing Property and Mechanism of LPSE

**Figure 8:**
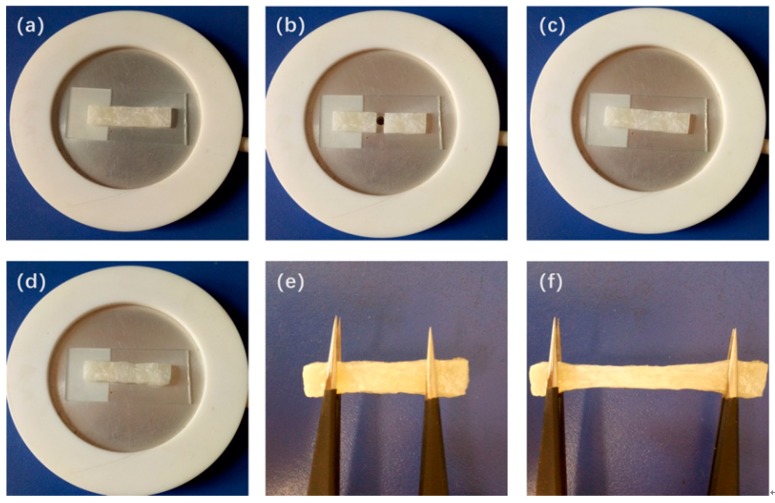
Self-healing process of PI-UPy observed by camera: (**a**) an initial elastomer sample; (**b**) the elastomer sample cut into two parts; (**c**) the separated two parts put together; (**d**) the elastomer sample heated on the hot-stage; (**e**) and (**f**) the healed elastomer sample stretched to show its strength.

**Figure 9:**
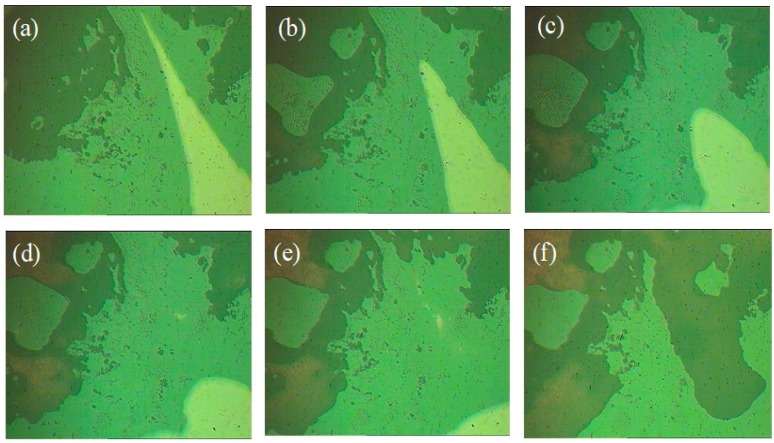
Self-healing process of PI-UPy observed with POM: (**a**) two parts put together; (**b**–**e**) the healing process of the elastomer sample; (**h**) the elastomer sample healed after heating.

Figure 8a–f show the self-healing process of PI-UPy. In order to characterize the self-healing ability of PI-UPy, the sample was cut into two parts with a knife, and then the pieces were put together. The system was heated at about 90 °C for some time, and it was gently stretched with tweezers. As expected, the elastomer could heal and became a continuum, and the elastomer could not be broken from the gap by simple stretching. Figure 9 presents the self-healing process of PI-UPy under POM. From the initial surface observation, the crack in the composite material was clearly visible. As the temperature increased, the crack of PI-UPy gradually disappeared in the composite material. Then, partial self-healing was successfully achieved. This may be due to the fact that PI-UPy melted at high temperatures, and the crack was filled by a mobile phase. In addition, after cooling, it became a solid state, and hydrogen bonds were gradually formed to achieve the self-healing process [37].

**Figure 10:**
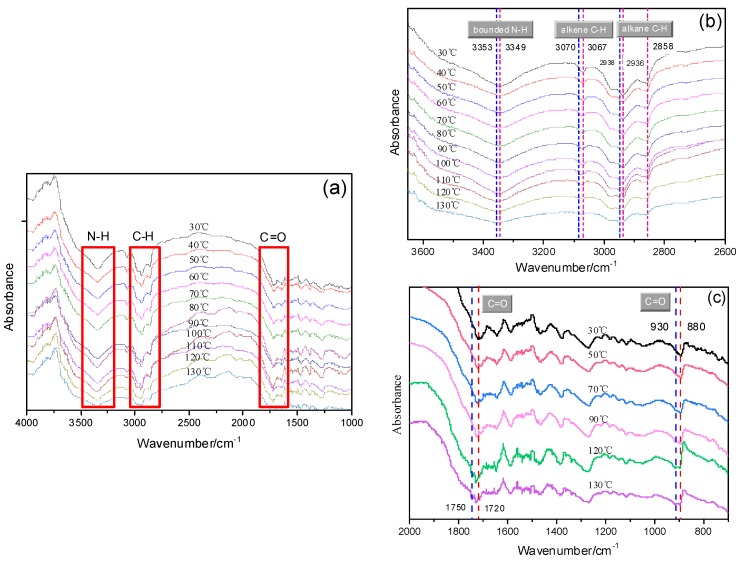
In situ variable-temperature infrared spectra of PI-UPy: (**a**) whole temperature ranges of initial elastomer sample; (**b**) blue shift of –NH_2_ and C–H; (**c**) blue shift of C=O.

To further reveal the hydrogen bonds in PI-UPy, the temperature-dependent FTIR spectra are presented in Figure 10. For original PI-UPy, the N–H, C–H, and C=O bonds appeared at 3349, 2800–3100, and 1720 cm^−1^, respectively, and these were ascribed to H-bonds in amide, methyl, and methylene groups (Figure 10a). With an increase in temperature, the wavenumbers gradually shifted to the region of higher frequency, and the absorption peaks widened. In particular, the characteristic peaks at 3349 cm^−1^ for N–H bonds, 3070 cm^−1^ for alkene C–H bonds, and 2858 and 2538 cm^−1^ for alkane C–H bonds became gradually apparent when the temperature was above 110 °C (Figure 10b). This may be because, when the temperature was above the melting point of the material, the hydrogen bonds in the material were destroyed. Moreover, higher wavenumbers reflected weaker hydrogen bonds [29]. It can be observed that N–H peaks were shifted from 3349 to 3353 cm^−1^, alkene C–H bonds were shifted from 3067 to 3070 cm^−1^, and alkane C–H bonds were shifted from 2936 to 2938 cm^−1^. These represent a type of blue shift which was related to the dissociation of the hydrogen bonds [38]. These results clearly proved that, with an increase in temperature, H-bonds in this composite became weaker. A similar phenomenon can also be seen from the change in stretching peaks for the C=O bonds (Figure 10c). With an increase in temperature, the center of this bond from amine groups was shifted from 1720 to 1750 cm^−1^ (stretching vibration) and from 880 to 930 cm^−1^ (bending vibration), and the intensity also decreased, illustrating that fewer and weaker H-bonded linkages were formed. The hydrogen bonds produced by the UPy groups in the polymer were not permanently crosslinked, and they dissociated and dissipated energy when the energy required was higher than that of the bond energy in the hydrogen bonds. The stress of the material was redistributed, and the hydrogen bonds in the material were further dissociated during this process. Therefore, the elastomer featured larger deformation variables and moduli when the stress was applied.

**Figure 11:**
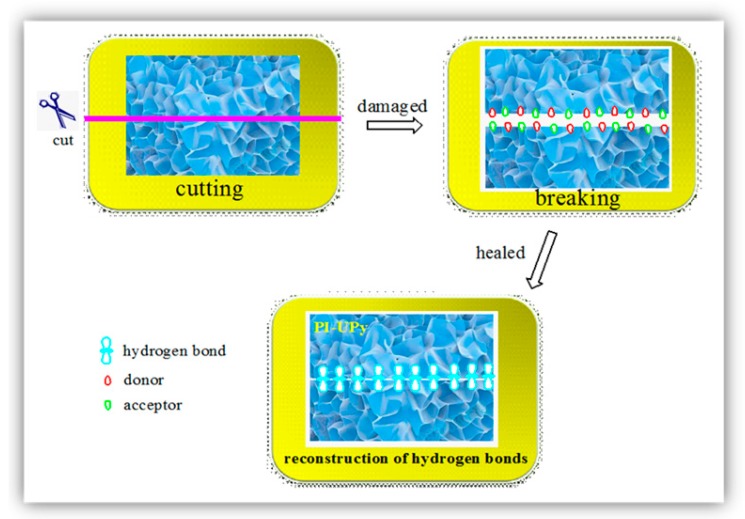
Schematic illustration of damage and self-healing process of PI-UPy.

Several self-healing mechanisms were reported in the literature, such as hydrogen bonding mechanisms, ionic bonding mechanisms, metal–ligand coordination mechanisms, and so on. These mechanisms all belong to the physical crosslinking category, and they attracted researchers’ attention during recent years [39]. Here, a possible hydrogen bonding mechanism is proposed to illustrate the excellent self-healing performance of PI-UPy, as shown in Figure 11. When PI-UPy was cleaved into individual parts, the physical interaction including the UPy dimer derived from the quadruple hydrogen bond was destroyed. Due to the reversibility of the hydrogen bonds, the damaged polymers could self-heal for a certain period of time after being put together. This indicated that crosslinking could lead to self-healing after being destroyed, which helps to prepare materials with good self-healing properties.

## 4. Conclusions

In this work, the anionic polymerization method was used to prepare a novel type of supramolecular elastomer, LSPE. Hydroxyl, isocyanate, and UPy groups were grafted at the ends of the backbones of polyisoprene which were covalently crosslinked. Thus, a supramolecular structure was formed, and higher bond energy was possessed by this novel elastomer. 

FTIR and ^1^H-NMR analysis showed that PI-UPy was successfully prepared due to the reaction between MIC and PI-NCO. TGA results showed that the addition of UPy facilitated the formation of intermolecular hydrogen bonds, and the thermal stability of the polymer was improved. Stress–strain curves demonstrated that a significant stress hardening phenomenon occurred after a certain deformation. The elasticity and stiffness of the elastomer were characterized by DMA. When the temperature was higher than 20 °C, the tan δ value also obviously increased, and this indicated that its damping properties were improved. The temperature-dependent FTIR spectra demonstrated that, with an increase in temperature, the wavenumbers gradually shifted to the region of higher frequency, and the absorption peaks widened. As expected, due to strong hydrogen bonds in the system, the elastomer featured self-healing behavior and became a continuous body at elevated temperatures. 

In summary, this toughened supramolecular elastomer concept based on multiple hydrogen bonds can also be applied to other elastomers such as polybutene and styrene butadiene rubbers.

## Figures and Tables

**Table 1:** The gel permeation chromatography (GPC) results of hydroxyl-terminated polyisoprene (PI-OH).

*M*_n_ (g/mol)	*M*_w_ (g/mol)	*M*_z_ (g/mol)	*M*_p_ (g/mol)	D (*M*_w_/*M*_n_)
48,290	53,450	60,850	47,150	1.107
